# Protocol for Bone Augmentation with Simultaneous Early Implant Placement: A Retrospective Multicenter Clinical Study

**DOI:** 10.1155/2015/589135

**Published:** 2015-11-24

**Authors:** Peter Fairbairn, Minas Leventis

**Affiliations:** ^1^Department of Periodontology and Implant Dentistry, School of Dentistry, University of Detroit Mercy, 2700 Martin Luther King Jr. Boulevard, Detroit, MI 48208, USA; ^2^Department of Oral and Maxillofacial Surgery, Dental School, University of Athens, 2 Thivon Street, Goudi, 115 27 Athens, Greece

## Abstract

*Purpose.* To present a novel protocol for alveolar bone regeneration in parallel to early implant placement.* Methods.* 497 patients in need of extraction and early implant placement with simultaneous bone augmentation were treated in a period of 10 years. In all patients the same specific method was followed and grafting was performed utilizing* in situ* hardening fully resorbable alloplastic grafting materials consisting of *β*-tricalcium phosphate and calcium sulfate. The protocol involved atraumatic extraction, implant placement after 4 weeks with simultaneous bone augmentation, and loading of the implant 12 weeks after placement and grafting. Follow-up periods ranged from 6 months to 10 years (mean of 4 years).* Results.* A total of 601 postextraction sites were rehabilitated in 497 patients utilizing the novel protocol. Three implants failed before loading and three implants failed one year after loading, leaving an overall survival rate of 99.0%.* Conclusions.* This standardized protocol allows successful long-term functional results regarding alveolar bone regeneration and implant rehabilitation. The concept of placing the implant 4 weeks after extraction, augmenting the bone around the implant utilizing fully resorbable, biomechanically stable, alloplastic materials, and loading the implant at 12 weeks seems to offer advantages when compared with traditional treatment modalities.

## 1. Introduction

According to the Branemark original protocol, implant placement was carried out 6 to 8 months after tooth extraction followed by a 3- to 6-month stress-free osseointegration period resulting in a long overall treatment time [1]. In an attempt to shorten the time frame between extraction and prosthetic delivery and to reduce cost, patient discomfort, and the number of surgical interventions, the immediate placement of implants at the time of tooth extraction has been proposed [2]. Other potential advantages with immediate implants are that the amount of bone loss at the extraction site might be reduced and optimal soft tissue aesthetics may be achieved [3]. On the other hand, there are some disadvantages with immediate implants such as the enhanced risk of infection and the lack of soft tissue closure [4, 5]. In order to overcome these potential problems early placement of implants has been proposed [2]. In this technique the clinicians wait 2 to 8 weeks before placing the implant to achieve some soft tissue healing and decrease the risk of infections [5].

The short-term survival rate of implant placement appears similar between immediate, early, and late approaches. However, at present there is little data on the success of immediate and early placement compared to late placement [2, 3, 5]. A few reviews evaluating the efficacy of immediate or early implants have been published over the years, but so far evidence is inconclusive [4–11].

With immediate or early implants it is possible that one or more bony walls of the postextraction socket are either partly or completely missing due to the preexisting inflammatory processes or damaged as a complication of the tooth extraction procedure. As a result, a portion of the implants could remain exposed due to hard tissue defect. Sockets with dehiscence defects may lack the potential for complete bone regeneration, and the risk of long-term complications may be increased with immediate or early implants placed at these sites [5]. However, several reports have shown that bone regeneration may be achieved in defective sites adjacent to immediate or early implants using a variety of bone augmentation techniques, such as autogenous bone grafts, bone substitutes, and guided bone regeneration with resorbable or nonresorbable barriers [4]. However, there is no enough reliable evidence supporting or refuting the need for augmentation procedures in parallel to immediate or early implant placement or whether any of the augmentation techniques is superior to the others [4, 5, 12].

When regenerating lost alveolar bone with the use of grafting materials, an important concern is the presence of residual particles, which might interfere with normal healing and bone-to-implant contact. The quality of the regenerated bone around immediate or early implants might be critical in determining the long-term function and stability of dental implants and the peri-implant tissues [13]. Beta-tricalcium phosphate (*β*-TCP) has a compressive strength similar to that of cancellous bone and undergoes resorption over a 6–18-month period being completely replaced by newly formed vital bone [14–18]. However, few studies to date have evaluated the long-term outcome of using *β*-TCP as grafting material simultaneously with implant placement into extraction sites [14, 19].

It would be of great benefit to investigate if completely resorbable* in situ* hardening alloplastic grafting materials could be used, without the need of membrane coverage, during early implant placement in a successful and predictable way. The purpose of the present study was therefore to assess the long-term survival rate of implants early placed into defective sockets with simultaneous bone grafting with* in situ* hardening *β*-TCP, following a standardized protocol.

## 2. Patients and Methods

This study reports a series of 497 patients treated according to the novel protocol, from August 2004 to July 2014. Patients were referred for consultation and treatment of nonsalvageable teeth due to root fractures, advanced caries, trauma, periodontitis, or failed endodontic treatment. All patients were treated in 2 private implantology clinics by 2 different clinicians. In the present study, only cases with defective buccal bone wall and need for bone augmentation in parallel to early implant placement were included. Patients with intact 4-wall postextraction sockets, with uncontrolled diabetes, alcoholics, and drug abusers were excluded, but smokers were included. All patients signed a letter of consent for the use of the alloplastic bone graft substitutes and implant placement.

After thorough clinical examination, periapical radiographs were taken. In 48% of the cases where additional information was required, a CBCT was prescribed.

In all cases the same standardized methodology was followed: Firstly, after local anaesthesia, teeth were “atraumatically” extracted without raising a flap. Extractions were facilitated by the use of periotomes and gentle elevation. Attention was given not to damage the surrounding soft and hard tissues. In cases of multirooted teeth, teeth were sectioned and removed in pieces. After extraction, the sockets were thoroughly curetted and debrided of inflammatory tissue, followed by rinsing with sterile saline. Postextraction sockets were allowed to heal by secondary intention.

After 4 weeks a site-specific full thickness flap was raised buccally using vertical releasing incisions, without including the papillae of the adjacent teeth. After flap elevation all granulation tissue was removed from the site and a tapered implant (Dio, Dio Co., Busan, Korea) was placed in the optimal position. After placing the cover screw, the site was augmented utilizing an* in situ* hardening resorbable alloplastic bone grafting material.

Fortoss Vital (Biocomposites, Staffordshire, UK) is a biphasic alloplastic bone graft consisting of *β*-TCP in a calcium sulfate (CS) matrix. This graft material has an increased negative isoelectric charge (Zeta Potential Charge [ZPC]) in an aqueous solution, which has been shown to upregulate the host response by attracting positively charged host bone morphogenetic proteins to the site. These in turn result in the increased presence of osteoblasts to the site for improved early bone regeneration. Fortoss Vital acts as a scaffold for bony proliferation as it is slowly resorbed by osteoclastic activity and substituted by living bone cells that grow directly in contact with the mineral. The product forms a simple to use, moldable cohesive paste that sets to form a hard, but resorbable, osteoconductive bone graft material.

Ethoss (Regenamed Ltd., London, UK) is a biphasic alloplastic grafting material consisting of *β*-TCP (65%) and CS (35%). When mixed with sterile saline, the material forms an easily handling moldable mass that hardens* in situ*.

No barrier membranes were used. The mucoperiosteal flap was repositioned and sutured without tension with resorbable 4-0 sutures (Vicryl, Ethicon, Johnson & Johnson, Somerville, NJ, USA). The sutures were removed after a 7-day healing period.

After 10 weeks a similar site-specific full-thickness flap was raised to access the cover screw. In 60% of the cases the stability of the implants was evaluated by resonance frequency analysis (Osstell ISQ, Gothenburg, Sweden). A healing abutment was placed and the flap was then sutured using 4-0 sutures (Vicryl Rapide, Ethicon, Johnson & Johnson, Somerville, NJ, USA). Lastly after allowing the soft tissue to mature for 2 weeks the final titanium abutment was placed and a cemented metal-ceramic restoration was fabricated.

## 3. Results

**Figure 1:**
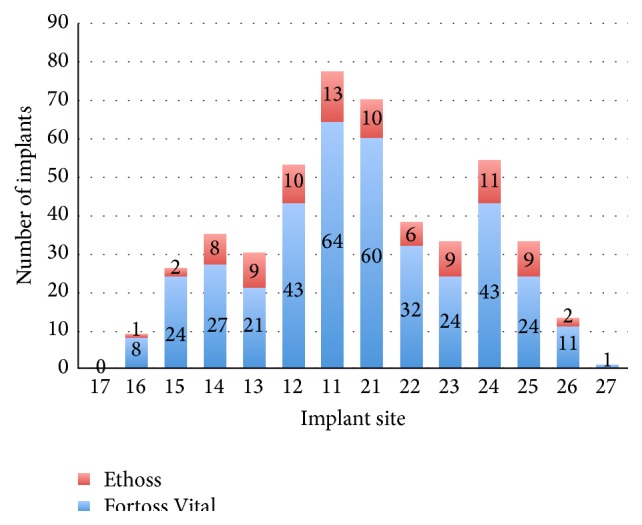
Implant distribution and grafting material used in maxilla.

**Figure 2:**
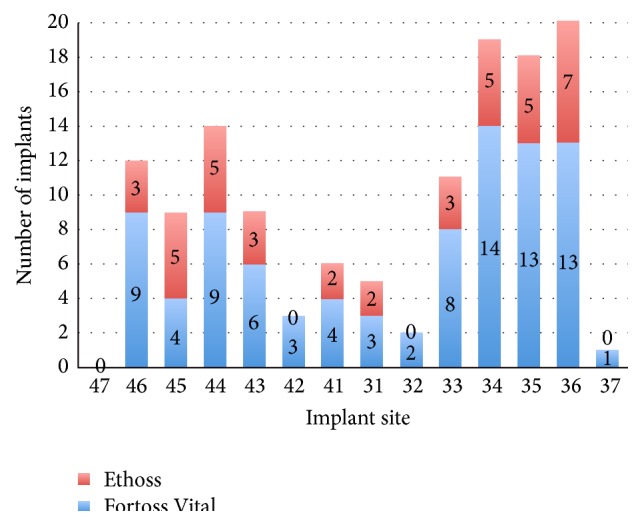
Implant distribution and grafting material used in mandible.

This retrospective study of 497 patients included 243 females (48.9%) and 254 males (51.1%) with mean age of 54.24 years (range 23 to 91). In total 601 implants were early placed in different locations according to the novel protocol, and, of the 601 sites, 471 (78.4%) were grafted with Fortoss Vital, and 130 (21.6%) were grafted with Ethoss. The implant distribution, in accordance with the grafting material used, is shown in Figures 1 and 2.

Of the 601 implants placed, 3 were lost before loading (2 grafted with Fortoss Vital and 1 with Ethoss) due to infection and granulation tissue development; and 3 implants (2 grafted with Fortoss Vital and 1 with Ethoss) were lost 1 year after loading, corresponding to an overall success rate of 99.0%.

Apart from the 6 lost implants, none of the patients experienced postoperative complications.

At reentry, 10 weeks after implant placement and grafting, the sites were filled with newly formed bone. Remnants of the grafting materials could be identified, be embedded, and be in continuity with the newly formed bone. In many cases, the regenerated bone was completely or partially covering the implant cover screw. Out of the 595 successful cases only 5 (3 grafted with Fortoss Vital and 2 grafted with Ethoss) needed minor additional grafting buccally with the same material in order to cover still exposed cervical implant threads, without compromising the final result. At this time point all implants were firmly integrated and ISQ measurements, when available, showed high (70–84) values.

All successful cases were loaded with cemented crowns and the pleasing esthetic outcomes were noted.

Follow-up radiological examinations with periapical X-rays (follow-up periods ranged from 6 months to 10 years, mean of 4 years) demonstrated stable peri-implant hard tissues.

**Figure 3:**
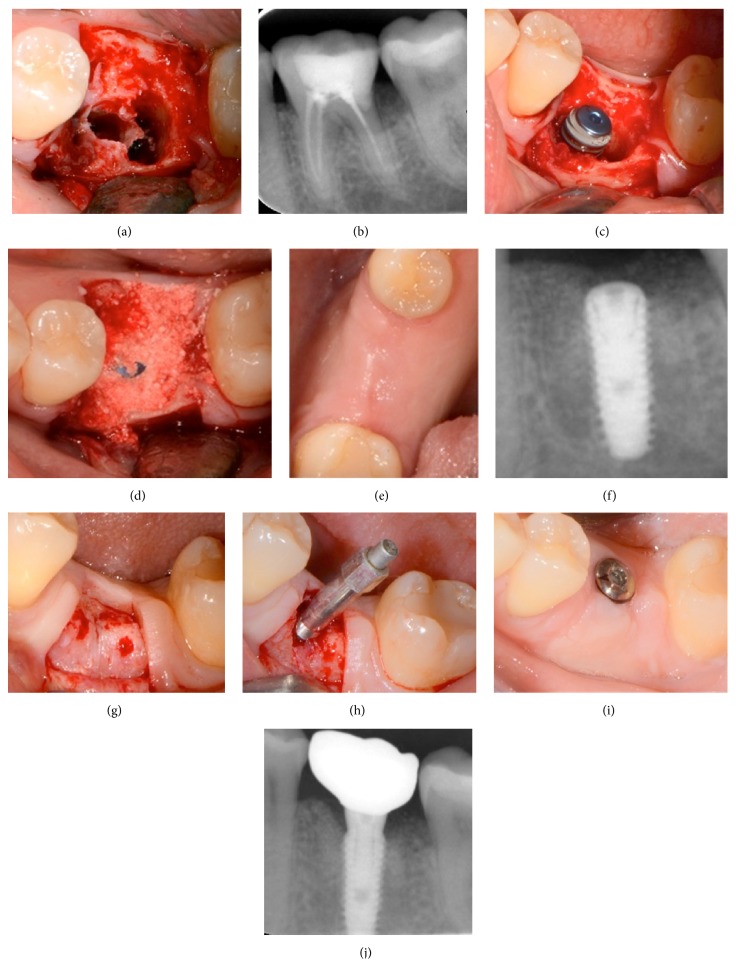
Case  1: a 47-year-old woman with crown and root fracture in the left mandibular first molar. (a) Clinical view of the site after thorough debridement of the socket. (b) Periapical X-ray of the nonrestorable tooth. (c) Implant placement at the correct 3D positioning. ISQ reading was 48. (d) Grafting with *β*-TCP/CS (Ethoss). (e) Clinical view after 10 weeks. (f) X-ray 10 weeks after implant placement and grafting showing the consolidation of the grafting material around the implant and new bone formation over the implant head and towards the adjacent interproximal heights of bone. (g) At reentry the site is filled with regenerated bone. Note the head of the implant covered by newly formed bone. (h) After removing the supernatant newly formed bone with a round burr implant stability is assessed (ISQ measurement: 78) revealing a significant increase through the 10-week healing period. (i) Maturation of the soft tissues 2 weeks after placement of the healing abutment. (j) X-ray 9 months after loading.

**Figure 4 fig4:**
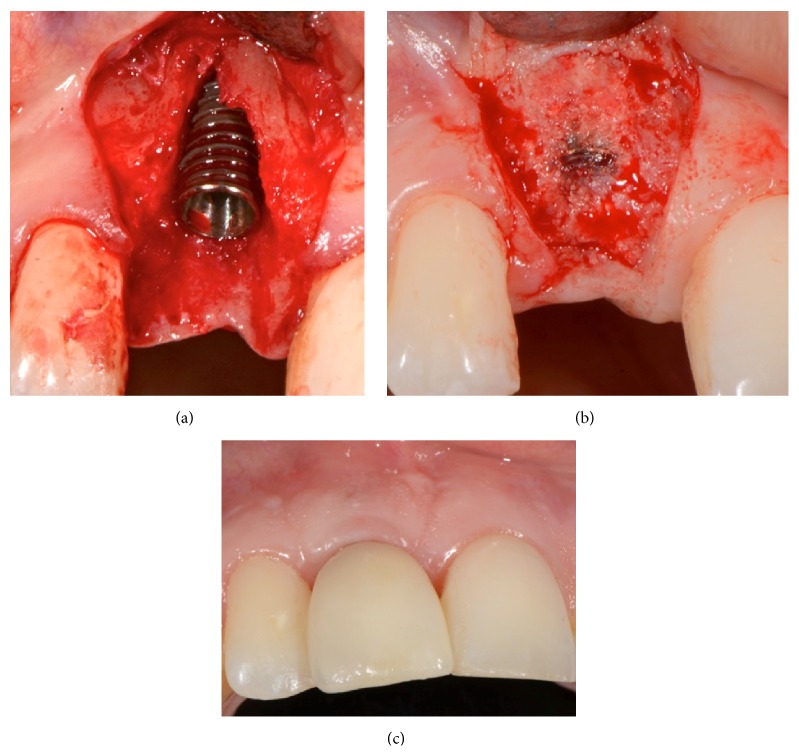
Case  2: a 28-year-old woman with root fracture in the maxillary right central incisor. (a) Implant placed at the optimum 3D positioning leaving a buccal dehiscence. (b) Reentry after 10 weeks revealing complete bone regeneration of the site. The head of the implant is partially covered by newly formed bone and the ridge is also significantly augmented laterally. ISQ reading was 75. (c) Six months after loading, excellent preservation of the buccal profile.

**Figure 5 fig5:**
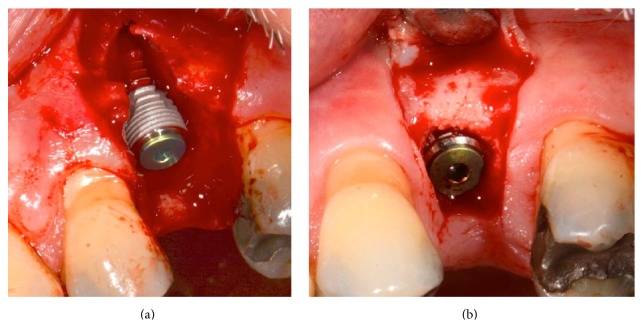
Case  3: a 62-year-old male with root fracture in the maxillary left second premolar. (a) Implant placed at the optimum 3D positioning with low initial stability, leaving a buccal dehiscence. (b) Reentry after 10 weeks showing excellent bone regeneration of the site. ISQ reading was 76.

**Figure 6:**
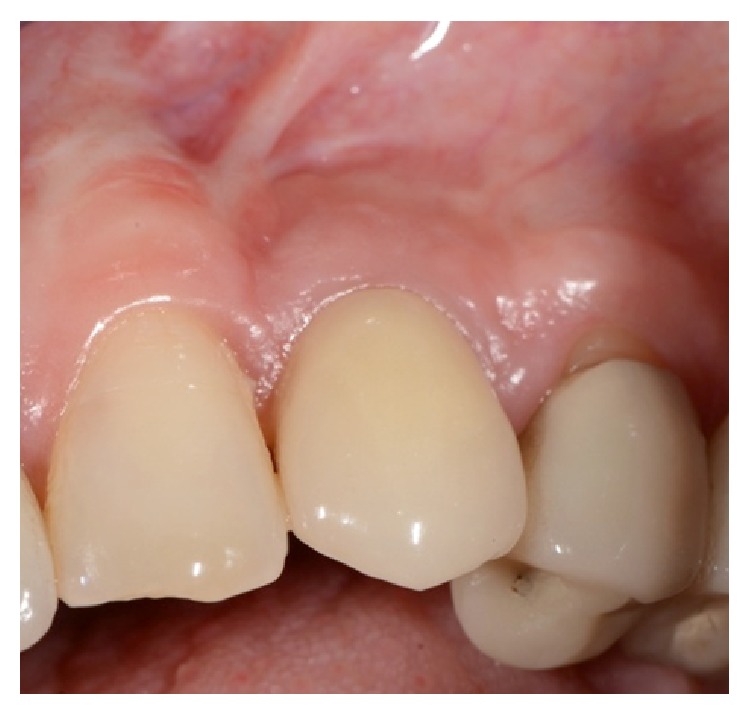
Seven-year follow-up clinical picture of a maxillary left canine case treated according to the protocol and grafted with Fortoss Vital.

Figures 3 [Fig fig4] [Fig fig5]–6 show 4 cases treated according to the proposed protocol.

## 4. Discussion

This report proposes a protocol for early implant placement and simultaneous bone augmentation in sites with dehiscence-type bone defects.

A potential advantage with early implantation compared to immediate placement seems to be the decreased risk of infections and associated implant failures. The findings of the present study support this hypothesis as from the 601 placed implants 4 weeks after extraction only 3 (0.5%) were lost due to infection during the healing period. The overall success rate in this study was 99.0%, higher than the success rate reported in the literature with regard to survival percentages ranging from 95% to 97.5% [7, 8, 20–24].

Although there is currently too little evidence to draw definitive conclusions [5], the literature suggests that the placement of dental implants at an early timing after tooth extraction may also offer advantages in terms of soft and hard tissue preservation, when compared with immediate or delayed protocols [7, 12, 20–26]. The survival rates presented in this case series study show stable functional outcomes in a follow-up period up to 10 years (mean of 4 years). In 595 cases the contour augmentation technique described in this protocol was able to regenerate the hard tissues around the implants, as observed at the 10-week postop reentry, and allow for long-term function and clinical survival of the implants.

The concept of bone augmentation with the use of xenogeneic bone graft and a resorbable barrier membrane in conjunction with early implant placement was carried out in several clinical studies with successful results [23, 24, 26, 27].

In contrast to the above augmentation protocols, in the present study a different rationale for bone augmentation in parallel with early implant placement was followed. In all cases the dehiscence-type bone defects were treated with resorbable biphasic alloplastic bone grafting materials composed of *β*-TCP and CS and no barrier membranes were used. Significant bone formation at the buccal aspect of the implants was demonstrated at reentry after 10 weeks and only in 0.8% of the cases additional grafting was needed in order to cover still exposed cervical implant threads. It seems that the biomechanical properties of the grafting materials used in this study fulfilled the main principles of successful bone regeneration of the alveolar bone, that is, exclusion of gingival tissue from the regenerating site and maintenance of a stable bacterial-free closed compartment [28]. The CS component of the grafting materials used is pyrogen-free and bacteriostatic, creating a nanoporous cell-occlusive membrane that prevents the early stage invasion of unwanted soft tissue cells and when mixed with other grafting materials enhances graft containment, making the mixture more stable and pressure resistant [29–31]. Adding CS to *β*-TCP produces an* in situ* hardening grafting material that binds directly to the host bone, maintains the space and shape of the grafted site, and acts as a stable osteoconductive scaffold [32, 33]. The improved stability throughout the graft material seems to further improve the quality of the bone to be regenerated due to reduced micromotion of the material, which may lead to mesenchymal differentiation to fibroblasts instead of osteoblasts [34]. It is known that micromovements between bone and any implanted grafted material prevent bone formation, resulting in the development of fibrous tissue [35]. A possible problem with particulate grafts like deproteinized bovine bone mineral might be the lack of stability of the grafting material at the recipient site. In such cases a resorbable membrane is needed to cover and stabilize the particulate grafting material [36].

In the present study the *β*-TCP/CS bone grafts were covered only with the mucoperiosteal flap. The 4-week healing period after the extraction enabled the production of adequate newly formed keratinized tissue, achieving tension-free primary closure and maintenance throughout the healing and regeneration phases. The no need for a barrier membrane in the proposed protocol significantly reduced the surgical time and cost and may be attributed to enhanced bone regeneration as the periosteum was not isolated from the grafted site. Periosteum has been shown to play a pivotal role in bone graft incorporation, healing, and remodeling, as it contains multipotent mesenchymal stem cells that are capable of differentiating into bone and cartilage and provides a source of blood vessels and growth factors [37, 38].

The profound bone regeneration shown after 10 weeks in the present study may also be explained by the biological properties that characterize alloplastic materials used. It has been found that *β*-TCP when covered with vascularized periosteum enhances osteoconduction and osteoblastic activity while resorbing simultaneously with the formation of new bone. There is also ongoing important evidence that TCP possesses high osteoinductive potential [14, 39–42]. Moreover, experimental research has shown that the addition of the resorbable CS to the graft significantly accelerates osteogenesis and increases calcification and the quantity of new bone in a shorter period of time [33, 43]. It is also important that the most intensive osteogenic activity during healing of extraction sites takes place between 4 and 8 weeks after extraction. Placement of the implant and the grafting material at 4 weeks after atraumatic extraction takes advantage of this enhanced host bone-healing environment [14, 44]. Also, it has been found that implant insertion increases bone metabolic activity at the site [45], further contributing to enhanced bone regeneration. Although a histological evaluation of the regenerated hard tissue was not performed in this study, it can be assumed that the bone defects around the implants have been repaired and finally filled with high quality vital bone free of residual graft particles.

There are concerns that bone grafting materials like *β*-TCP and CS that are fully resorbed in a short timeframe may contribute to site collapse [15, 16, 33, 42, 46]. Early loading of the implants after 12 weeks, as proposed in the present protocol, may further enhance the metabolic activity and trigger the remodelling of the regenerated labial bone [44]. Assuming that the newly formed hard tissue at the facial aspect of the implant is vital bone with no residual graft particles, it can be concluded that it adapted successfully to the transmitted occlusal forces according to Wolff's law, became stronger to resist to the type of loading, and thus maintained long-term the bone function [47, 48].

## 5. Conclusions

The results of this study suggest that this novel standardized protocol allows successful and predictable long-term successful functional outcomes regarding alveolar bone regeneration and implant rehabilitation. The concept of placing the implant 4 weeks after extraction, augmenting the bone around the implant utilizing only fully resorbable, biomechanical stable, alloplastic *β*-TCP/CS materials, and loading the implant at 12 weeks seems to offer advantages when compared with traditional treatment modalities. Additional studies are needed in order to confirm the present findings.
